# Recent Perspectives on Gene-Microbe Interactions Determining Predisposition to Otitis Media

**DOI:** 10.3389/fgene.2019.01230

**Published:** 2019-11-26

**Authors:** Rahul Mittal, Sebastian V. Sanchez-Luege, Shannon M. Wagner, Denise Yan, Xue Zhong Liu

**Affiliations:** ^1^Department of Otolaryngology, University of Miami Miller School of Medicine, Miami, FL, United States; ^2^Department of Pediatrics, University of Miami Miller School of Medicine, Miami, FL, United States; ^3^Dr. John T. Macdonald Foundation, Department of Human Genetics and John P. Hussman Institute for Human Genomics, University of Miami Miller School of Medicine, Miami, FL, United States

**Keywords:** otitis media, genetics, microbiome, gene-microbe interactions, twin studies, mouse models

## Abstract

A comprehensive understanding about the pathogenesis of otitis media (OM), one of the most common pediatric diseases, has the potential to alleviate a substantial disease burden across the globe. Advancements in genetic and bioinformatic detection methods, as well as a growing interest in the microbiome, has enhanced the capability of researchers to investigate the interplay between host genes, host microbiome, invading bacteria, and resulting OM susceptibility. Early studies deciphering the role of genetics in OM susceptibility assessed the heritability of the phenotype in twin and triplet studies, followed by linkage studies, candidate gene approaches, and genome-wide association studies that have helped in the identification of specific loci. With the advancements in techniques, various chromosomal regions and genes such as *FBXO11*, *TGIF1*, *FUT2*, *FNDC1*, and others have been implicated in predisposition to OM, yet questions still remain as to whether these implicated genes truly play a causative role in OM and to what extent. Meanwhile, 16S ribosomal RNA (rRNA) sequencing, microbial quantitative trait loci (mbQTL), and microbial genome-wide association studies (mGWAS) have mapped the microbiome of upper airways sites and therefore helped in enabling a more detailed study of interactions between host polymorphisms and host microbiome composition. Variants of specific genes conferring increased OM susceptibility, such as *A2ML1*, have also been shown to influence the microbial composition of the outer and middle ear in patients with OM, suggesting their role as mediators of disease. These interactions appear to impact the colonization of known otopathogens (*Streptococcus pneumoniae*, *Haemophilus influenzae*, and *Moraxella catarrhalis*), as well as *Neisseria*, *Gemella*, *Porphyromonas*, *Alloprevotella*, and *Fusobacterium* populations that have also been implicated in OM pathogenesis. Meanwhile, studies demonstrating an increased abundance of *Dolosigranulum* and *Corynebacterium* in healthy patients compared to those with OM suggest a protective role for these bacteria, thereby introducing potential avenues for future probiotic treatment. Incorporating insights from these genetic, microbiome, and host-pathogen studies will allow for a more robust, comprehensive understanding of OM pathogenesis that can ultimately facilitate in the development of exciting new treatment modalities.

## Introduction

Otitis media (OM) is one of the most common childhood diseases. With prevalence reaching as high as 5,000,000 annually in the United States, OM is a leading cause for pediatrician office visits and antibiotic prescriptions each year (Kaur et al., 2017). The associated medical expenditures account for as high as $4.1 billion, contributing to a significant healthcare utilization burden for both the healthcare system and patients (Gates, 1996; Bondy et al., 2000; Coticchia et al., 2013; Ahmed et al., 2014; Kaur et al., 2017). OM primarily affects children between the ages of 6 and 24 months, with 80–90% infants likely to experience OM disease at least once before reaching school age despite the introduction of the heptavalent pneumococcal conjugate vaccine in 2000 and its tri-decavalent counterpart in 2010 (Pichichero et al., 2008; Harmes et al., 2013; Kaur et al., 2016; Kaur et al., 2017). With resistance to standard antibiotic treatment continuing to increase steadily, the need for a more comprehensive understanding of OM pathogenesis and potential treatment modalities has become an immediate priority for pediatric and otolaryngology clinicians.

The specific diagnostic classification of OM varies according to its clinical presentation, which is characterized by specific signs and symptoms, disease progression, and the effectiveness of treatment. OM can be classified as acute otitis media (AOM) or otitis media with effusion (OME) when findings include an accumulation of fluid (middle ear effusion) behind the tympanic membrane (Harmes et al., 2013; Schilder et al., 2016). According to the latest guidelines, a diagnosis of AOM requires a moderate to severe bulging of the tympanic membrane, otorrhea, and ear pain with erythema; in contrast, these symptoms are often absent in OME (Lieberthal et al., 2013; Siddiq and Grainger, 2015). When AOM/OME persists despite treatment with antibiotics and/or surgical methods, chronic otitis media (COM), chronic otitis media with effusion (COME), or chronic suppurative otitis media (CSOM) may be indicated. COM is defined as recurrent infection of the middle ear with dry tympanic membrane perforation. COME shares a similar clinical presentation but with the addition of continuous serous drainage, while CSOM features purulent drainage leaking from a perforated tympanic membrane or ventilation tube (Mittal et al., 2015; Schilder et al., 2016). Chronic OM etiologies tend to be more serious for patients, as frequent and/or persistent dysfunction of the Eustachian tube may lead to significant hearing loss, thereby negatively impacting language acquisition and behavioral development in children (Cripps and Kyd, 2003; Santos-Cortez et al., 2016; Walker et al., 2019).

In many clinical cases, the manifestations of OM may overlap and co-occur. Therefore, the diagnostic distinctions between the many OM etiologies are important as they help in determining the adequate course of treatment and expected duration of disease. Some physicians utilize watchful waiting even with diagnosed OM cases to allow for the activation and response of the patient’s immune response that may help in the clearance of infection. Symptomatic therapy is indicated in the vast majority of OM cases, with antibiotic treatment regimens only appropriate when cases are persistent. Surgical methods such as tympanostomy tube or grommet insertion are implemented when case recurrence is especially severe (Pichichero et al., 2001; Kozyrskyj et al., 2010). Beyond guiding treatment, however, diagnostic classifications have also been linked to particular offending pathogenic microorganisms despite the fact that obtaining a culture-confirmed diagnosis is not routine in the clinical setting. *Streptococcus pneumoniae*, non-typeable *Haemophilus influenzae* (NTHi), and *Moraxella catarrhalis* are the three main otopathogens known to cause AOM worldwide (Casey et al., 2010; Casey et al., 2013; Kaur et al., 2017; Lappan et al., 2018). International epidemiological studies of Latin American and Caribbean populations have also confirmed *S. pneumoniae* and NTHi as the most frequent AOM bacterial pathogens (Bardach et al., 2011). In addition to these known and culturable otopathogens, a growing body of research has helped highlight how other bacterial, fungal, and/or viral species differentially contribute to the pathogenesis of various OM subtypes (Chonmaitree et al., 2008; Pettigrew et al., 2011; Chonmaitree et al., 2016). For example, clinical and animal studies both demonstrate how inflammation and microbiome disruption of an initial viral and/or bacterial infection of the upper respiratory tract often contributes to the pathogenesis of AOM (Coticchia et al., 2013; Schilder et al., 2016; Dewan et al., 2019). Progression of AOM to COM is largely believed to be due to the complex interactions between bacterial, environmental, and host factors. *Pseudomonas aeruginosa* and *Staphylococcus aureus* (Mittal et al., 2015; Mittal et al., 2019) are the most common pathogens associated with CSOM. However, our current knowledge about COM, especially CSOM, is still very limited and further studies are warranted to understand the pathogenesis of disease.

Early studies attempted to identify the microbiological causes of OM that were limited to culturable bacteria. However, the expansion of 16S ribosomal RNA (rRNA) sequencing and other culture-independent methods have allowed for the identification of microorganisms within the nasopharynx (NP) and middle ear (ME) that play an important role in both resident microbiota and intruding pathogenic populations (Broides et al., 2009; Pichichero, 2009; Kaur et al., 2010). The advancement of these bioinformatic and genomic detection methods have allowed researchers to incorporate insights from the host genome and microbiome that have both deepened and complicated our knowledge of OM pathogenesis. Decades of research have helped uncover links between host genes and OM susceptibility, but our expanding repository of microbiome literature across many fields has introduced new questions regarding the host microbiota’s role in pathogenesis, progression, and persistence of many disease etiologies. For instance, recent studies exploring the interplay between individual genes and microbiome composition in the gastrointestinal system, such as the gene-microbe interaction found to drive the development of Crohn’s disease-like colitis in mice, suggesting the potential of this interdisciplinary approach for future OM research (Caruso et al., 2019). The goal is that these insights will lead to the development of novel treatment modalities such as probiotic therapies, thereby addressing concerns of growing antibiotic resistance amidst continuously high OM disease burden (Lappan et al., 2018; Mittal et al., 2018). In this article, we describe the recent developments in OM research that elucidate the interactions between host genes, microbiome composition, and OM pathogenesis, and we identify relevant gaps in understanding that should we believe should be prioritized in future research.

### Genetics of Otitis Media

#### Twin Studies

The first studies to assess the role of genetics in acquiring recurrent OM were mainly twin studies (**Table 1**). One of these initial studies was a retrospective study comprising of 2,750 pairs of Norwegian twins born between 1967 and 1974 (Kvaerner et al., 1997). A series of eight structural equation models were constructed to estimate genetic effects, individual environmental effects, common familial environment effects in males, and dominance effects in females on the predisposition to develop OM (Kvaerner et al., 1997). After selecting a model that best fit the data, it was found that heritability of OM was estimated at 45 and 75% in males and females, respectively (Kvaerner et al., 1997). It is important to note that, despite common misinterpretation, these heritability values do not suggest that OM is 45 or 75% caused by genetics. Instead, the heritability values suggest that 45 and 75% of the variability in developing OM can be attributed to genetic differences in the populations studied. Twin studies are vital in guiding further scientific research to study the role of genetics in OM. A few other important twin studies are described below.

**Table 1:** Significant heritability values from various twin and triplet studies.

Heritability	Ages affected	Case definition	Reference
Males: 0.45 Females: 0.75	0–7	Individuals with recurrent ear infections before 7 years old	Kvaerner et al., 1997
0.73	0–2	AOM: presence of effusion, at least one symptom (fever, otalgia, or irritability), and one sign of inflammation (erythema, bulging/fullness, or otorrhea) OME: presence of middle ear effusion without symptoms of AOM	Casselbrant et al., 1999
0.72	0–5	AOM: presence of effusion, at least one symptom (fever, otalgia, or irritability), and one sign of inflammation (erythema, bulging/fullness, or otorrhea) OME: presence of middle ear effusion without symptoms of AOM	Casselbrant et al., 2004
0.57	0–4	OM: earache, ears leaking pus/mucus, pulling or scratching ears, and red or sore ears	Rovers et al., 2002

Another study, a prospective twin and triplet study, investigated the genetic component of recurrent ME effusion and AOM (Casselbrant et al., 1999) (**Table 1**). From 1982 to 1995, 168 same-sex twin pairs and 7 same-sex triplet sets younger than 2 months old were recruited for the study mainly from Magee-Women’s Hospital in Pittsburgh, PA (Casselbrant et al., 1999). Only same-sex twin and triplet sets were included in the study due to differences in incidence of OM between males and females. From the 168 twin and triplet sets first recruited, 126 were followed for 2 years and any episodes of ME effusion or AOM were documented. From these 126 twin and triplet sets, the estimated degrees of discordance for three or more episodes of ME effusion were 0.04 and 0.37 for monozygotic and dizygotic twins, respectively. For an episode of AOM, the estimates of discordance were 0.04 in monozygotic twins and 0.49 in dizygotic twins. These discordance values imply that monozygotic twins shared similar presence of illness compared to dizygotic twins, who did not become ill in conjunction with each other as often. These results suggest genetics play some role in susceptibility of the illness since monozygotic twins showed more concordance with disease than dizygotic twins. Lastly, the heritability of recurrent ME effusion was estimated at 0.73 after 2 years, which suggests genetics plays a significant role in the individual variation of OM cases. In 1987, researchers altered the protocol to extend the follow-up period to 5 years (Casselbrant et al., 2004). Eighty-three sets of twins or triplets were followed for 5 years, and the heritability of recurrent ME effusion within 5 years of life was estimated at 0.72, further suggesting genetics may play a large role in susceptibility to OM (Casselbrant et al., 2004).

To further understand the extent of genetic predisposition for OM, the Twin Early Development Study, a longitudinal study of same-sex twins born in England and Wales in 1994, was conducted (Rovers et al., 2002). Unlike previous studies, this study set out to estimate the heritability of different manifestations and symptoms of OM compared to those of chronic airway blockage, which frequently accompanies persistent effusion (Rovers, 2002). The researchers followed 1,373 twin pairs for 2, 3, and 4 years, assessing the occurrence of OM symptoms (earache, ears leaking pus or mucus, pulling or scratching ears, and red or sore ears) and chronic airway blockage symptoms (breathing through the mouth and snoring or snorting in sleep). When the OM and the chronic airway blockage symptoms were combined, the heritability was estimated at 0.49, 0.66, and 0.71 for ages 2, 3, and 4, respectively. When tested separately, the heritability of OM symptoms averaged over all years was estimated to be 0.57, and the effect of twin shared environment was 0.18. The chronic airway blockage symptoms when tested alone showed higher heritability (0.72) and a lower effect of twin shared environment (0.10) compared to those of OM. This investigation elucidates the importance of separating the symptoms of OM from those of chronic airway blockage instead of considering the two as undifferentiated OM.

As briefly mentioned before, these twin studies, although a good starting point for investigating the role of genetics in OM, provide minimal evidence compared to other studies of genetic causation or predisposition to developing OM. The main reason for the poor level of evidence is that a twin study only provides information about the role of genetics in the variation of the disease in a population. Heritability values cannot be used at the individual level to declare that a specific disease is caused by genetic factors some percentage of the time. The other issue with twin studies is that they provide no insight as to the specific genetic loci that may be implicated in OM, only a general sense of genetic heritability. It is important to note that the heritability measures are applicable/specific only to the cohort in which they are estimated.

### Linkage Studies

Besides twin investigations, there has been interest in investigating genetic determinants of chronic and recurrent OM across the entire genome using linkage studies (Daly et al., 2004) (**Table 2**). In a linkage analysis, researchers assess the statistical linkage of segments of the genome with the phenotype of interest among a series of families. One study recruited individuals who received tympanostomy tube surgery to treat chronic or recurrent OM and their families (Daly et al., 2004). From 133 families, they acquired 591 DNA samples, 238 of which were affected by chronic or recurrent OM, for genetic screening. The DNA samples were then screened for 404 fluorescent microsatellite markers and analyzed by single- and multipoint nonparametric linkage (NPL). The single-point NPL analyses suggested significant evidence of linkage on chromosome 10q26.3 at marker *D10S212* (LOD 3.78) and suggestive evidence of linkage on chromosome 19q13.43 at marker *D19S254* (LOD 2.61). The multipoint NPL analyses suggested strong evidence of linkage on chromosome 19q near marker *D19S254* (LOD 2.53). Interestingly, the multipoint NPL analyses showed decreased linkage on chromosome 10q near marker *D10S212* (LOD 1.64). Conditional analyses were then performed on samples that supported linkage on 10q and 19q in order to potentially increase other significant regions of the genome (Daly et al., 2004). The conditional analyses revealed linkage for chronic or recurrent OM on a region of chromosome 3p [conditional (10q) LOD 2.43; conditional (19q) LOD 1.84; unconditional LOD 0.60]. These analyses suggest that there exists an interaction between several regions of the genome that plays a role in the risk of chronic or recurrent OM.

**Table 2:** List of chromosomes implicated in otitis media based on various nonparametric linkage analyses.

Chromosome	LOD score	Marker	Case definition	Reference
10q26.3^*^	3.78	*D10S212*	At least two data sources indicated positive results for ear examination, tympanogram, self-reported history, and/or medical record OR 1 data source indicated positive results for above findings and current middle-ear findings presumptive of COME/ROM history	Daly et al., 2004
19q13.43^*^	2.61	*D19S254*		
**19q**	2.53	Near *D19S254*		
**10q**	1.64	Near *D10S212*		
**3p^a^**	2.43	NA		
**3p^b^**	1.84	NA		
**3p^c^**	0.6	NA		
**17q12**	2.83	NA	Insertion of tympanostomy tube at least once for recurrent/persistent OM	Casselbrant et al., 2009
**6p25.1**	2.25	NA		
**19**	3.75	63.4 mb	Tympanostomy tube insertion for COME/ROM, presence of OM sequelae, and/or abnormal middle ear mechanics	Chen et al., 2011

*Single-point nonparametric linkage (NPL) analyses; all other linkages found from multipoint NPL analyses. ^a^Conditional analysis with 10q. ^b^Conditional analysis with 19q. ^c^Unconditional analysis (not significant). NA, not analyzed.

Another study also attempted a linkage analysis to further identify possible genes that increase the risk of OM (Casselbrant et al., 2009). The researchers recruited families with two or more full siblings who received tympanostomy tube insertions due to a history of OM. Four hundred and three Caucasian families (1,431 individuals) and 26 African American families (75 individuals) were genotyped. The researchers carried out an NPL analysis using 8,802 single-nucleotide polymorphisms (SNPs) on the larger Caucasian data set, at first by itself and then in conjunction with the African American data set. When analyzed by itself, the Caucasian data set suggested strong linkage on chromosome 17q12 (LOD 2.83) and 6p25.1 (LOD 2.25). There were also three other suggestive linkages on 10q22.3, 7q33, and 4p15.2, but these were not as significant as the linkage on 17q12. When the data sets were combined, the peaks on chromosomes 17, 6, and 4 became less significant, but the linkage on chromosome 10q22.3 became more significant. The authors also suggest that these linkage signals are near previously implicated genes *SFTPA2*, *IFNG*, and *TNF* (Casselbrant et al., 2009).

To further localize significant linkage signals, another study set out to fine map chromosome 19, the chromosome previously implicated in Daly et al. (2004) (Chen et al., 2011). The researchers recruited individuals who received tympanostomy tube surgery to treat chronic or recurrent OM and their families. The participants included all subjects from the initial study and an additional six new families (a total of 607 individuals from 139 families). The researchers performed an NPL analysis of 1,091 SNPs, the majority of them were from chromosome 19. The NPL analysis suggested significant linkage on chromosome 19 at position 63.4 mb (LOD 3.75) with LOD-1 support interval between 61.6 and 63.8 mb. This region of the genome contains over 90 known genes and includes several genes implicated in inflammatory and immune processes (Chen et al., 2011).

These linkage analysis studies were large leaps forward in illustrating the potential genetics of OM because they isolated specific genomic sequences that statistically linked with cases of OM. Despite these promising findings, though, linkage analysis has its limitations. In linkage analysis, there can be a significant increase in the rate of false positives and a reduction of statistical power (Ferreira, 2004). Therefore the results of linkage studies should be evaluated with caution. In an attempt to overcome the drawbacks of linkage analysis studies, researchers focused their efforts in candidate gene approach studies to test the statistical association between specific genes and/or markers implicated in OM from previous studies and cases of OM in a population.

### Otitis Media in Mouse Models

The mouse model is a useful tool to study and identify the genes relevant to OM. There are genes that are essential for the maintenance of the ME epithelial cell integrity and health and mutations/deletion in these genes can determine predisposition to OM: 1) *Tgif1*—it functions as a negative regulator of the transforming growth factor beta (TGFβ) signaling pathway. *Tgif1* mutant mice have significantly raised auditory thresholds due to a conductive deafness arising from OM (Tateossian et al., 2013). 2) *Phex*—the mutation in the *Phex* gene primarily upregulates the expression level of the *Fgf23* gene in the MEs and is linked to predisposition to OM in mice (Han et al., 2012). *Fgf23* mutant mice have also been shown to have mixed hearing loss and ME malformation (Lysaght et al., 2014). 3) *Oxgr1*—the *Oxgr1* gene encodes oxoglutarate receptor 1. The ligand for OXGR1 is believed to be involved in the regulation of vascular endothelial growth factor, an important inducer of angiogenesis and vascular permeability. Kerschner et al. (2013) showed presence of inflammatory cells, changes in the mucosal epithelium, and ME fluid in *Oxgr1* deficient mice. 4) *Mcph1*—the *Mcph1*-deficient mice had mild to moderate hearing loss (around 70% penetrance). Other defects of *Mcph1*-deficient mice included small skull sizes, increased micronuclei in red blood cells, increased B cells, and ocular abnormalities (Chen et al, 2013). 5) *Lmna*—*Lmna* mutant mice have profound early-onset hearing deficits and abnormal positioning of the Eustachian tube accompanied by OM (Zhang et al., 2012).

There is also evidence of the involvement of signaling pathways and inflammatory factors involved in OM. *Pai-1* knockout mice showed significant pathological changes of tympanosclerosis (Shin et al., 2014); mice deficient in the *C5a* gene have been shown to have reduced levels of IL-6, mKC, and MCP-1, in association with decrease in inflammatory cell recruitment, mucosal inflammation, and bacterial clearance (Tong et al., 2014); mice lacking the *IL-17A* gene, encoding interleukin 17A (IL-17A), a neutrophil inducing factor, were associated with abnormal recruitment and apoptosis of neutrophils (Wang et al., 2014).

### Mouse-To-Man Candidate Gene Study

All cohorts for OM reported thus far have low power to detect common genetic risk factors for disease and have low resolution. The specific genetic susceptibility loci for OM can be identified, however, through genome-wide or candidate gene association studies. Candidate gene studies can theoretically overcome these issues of low power and resolution by focusing directly on the association between disease and genetic variants with strong support for their involvement in the biology. Through candidate gene association studies, efforts have been made to test the association of chronic OM with specific genetic loci that have been identified as potential risk loci of OM in mouse models. In candidate gene studies, the frequencies of a genetic marker are compared between affected study subjects and control subjects. The control subjects can be unrelated healthy controls (case-control study) or healthy siblings or other family members (family study). But with the candidate gene approach, ascertaining relevant loci for testing can be difficult and thus are likely to miss many causal regions. Increasingly, genetically altered mouse models, such as those discussed earlier, have been used to study OM because of the technology available to genetically alter this species and translate the analysis of suspected genes to the human model.

Bhutta et al. (2017) performed a genetic association study on a large cohort of children with COME, and tested SNPs at candidate loci derived from four genetically altered mouse models that have identified *Fbxo11*, *Evi1*, *Tgif1*, and *Nisch* as potential risk loci for chronic OM. Of the 1,296 families analyzed, evidence of association was found at rs881835 and rs1962914 at the *TGIF1* locus, with odds ratios of 1.4–1.6. There was a weaker association with rs10490302 and rs2537742, two SNPs within *FBXO11*, with odds ratios of around 1.2. All these SNPs are located in intronic regions. However, no evidence of association with the loci *EVI1* or *NISCH* was detected. Both the *TGIF1* and *FBXO11* loci are thought to be involved in TGF-β signaling, which implies that this pathway may be critical in the transition from acute to chronic ME inflammation. However, Bhutta et al. (2017) reported that these results failed to replicate in a case-control cohort in Finland of 402 children with chronic OM with effusion and 777 control participants.

In previous studies, Rye et al. (2011), in an analysis of 434 families predominantly suffering from recurrent AOM (p = 0.009) from Western Australia, showed an association with the major A allele at SNP rs330787 at *FBXO11*, with replication of this finding in an independent cohort (p = 6.9 × 10–6, OR = 1.55). Segade et al. (2006) also reported nominal evidence of *association to the* SNP rs2134056 (p = 0.017) at *FBXO11* in their cohort of 142 families from the US (with a mixed OM phenotype). The *TGIF1* locus, found to be associated with risk of COME, has not been evaluated in any previous genetic association study. *EVI1* and *NISCH* were not associated with OM (Sale et al., 2011).

The data therefore indicate that regulation of the TGF-β pathway may be critical for the development of persistent inflammation in the ME. TGF-β has already been reported as a key regulator of inflammatory response, through its effects on chemotaxis, activation, and survival of lymphocytes, natural killer cells, dendritic cells, macrophages, mast cells, and granulocytes (Yoshimura et al., 2010).

Other studies have attempted to isolate particular genetic loci associated with susceptibility to OM using a candidate gene association approach. What follows is a brief description of various other studies and their findings. A candidate gene study on 15 genes with single-nucleotide polymorphisms (SNPs) in 142 families with 618 subjects was conducted by Sale et al. (2011). Nominal genetic association was found for *MUC5AC* [rs2735733, *P* = .002, odds ratio (OR) = 0.646; rs7396030, *P* = .049, OR = 1.565; rs2075859, *P* = .041, OR = 0.744]. A trend toward association was also found in three other SNPs at three genes: *SCN1B* (rs810008, *P* = .013), *SFTPD* (rs1051246, *P* = .039), and *TLR4* (rs2770146, *P* = .038). Rye et al. (2013) have shown functional evidence for a role for *SLC11A1* in susceptibility to OM and have studied the candidate gene in 660 affected children from 531 families in a case/pseudo-control study. The best SNP association was detected at the rs2776631 (*P* = .025). Furthermore, haplotype analysis revealed the 3_C_C_G haplotype (rs34448891_rs2276631_rs3731865_rs2695343) to be more common in the affected individuals (*P* = .0008). MacArthur et al. (2014), in a candidate gene study of 192 SNPs from eight genes on 100 cases and 79 controls, reported that 8 SNPs on four genes (*TLR4*, *MUC5B*, *SMAD2*, *SMAD4*) showed a potential trend toward association (*P* unadjusted = 0.007, rs10116253 on *TLR4*). It is important to note, though, that these eight SNPs did not show significant association after correcting for multiple statistical testing (*P* adjusted = 0.625, rs10116253 on TLR4). Also, an increased risk for OM proneness was found associated with the *CX3CR1* gene (Thr280Met, OR = 6.23, *P* = .038) in a study of 21 SNPs as risk factors for upper respiratory tract infections, OM, and OM proneness in 653 children (Nokso-Koivisto et al., 2014). Ilia et al. (2014) conducted studies in a Greek cohort of 96 children on eight SNPs of five cytokine genes, that have demonstrated that *IL-10* (−592, −819, and −1082) and *TGF*β (codon 10C > T) were associated with an increased number of acute OM episodes (*P* < .001, *P* < .001, *P* < .001, and *P* = .002, respectively); *IL-10* (−1082) and *TGF*β (codon 10C > T) were also associated with a later onset of AOM (*P* = .007 and *P* = .0039, respectively). Gessner et al. (2013) reported in 1,032 study subjects from Alaska that individuals homozygous for the arctic variant of *CPT1A* (c.1436C > T) demonstrated a trend to more likely have OM (OR = 3.6, 95% confidence interval: 1.4–8.9). Hafren et al. (2015)performed a candidate gene study with 53 SNPs on 624 study subjects with recurrent acute OM and/or chronic OM with effusion and 778 control subjects. The positive result for *TLR4* (rs5030717, OR = 1.33, *P* = .003) was further investigated by a tagging SNP analysis and has resulted in the identification of two additional SNPs (rs1329060 and rs1329057). There was an increased association among patients with a more severe phenotype: those with OM starting before the age of 6 months (OR = 2.42, *P* = .0005, for rs1329060) and those with repeated insertions of tympanostomy tubes (OR = 1.65, *P* = .00004 for rs1329060). The finding was replicated in a Finnish OM cohort of 205 children (s1329060, OR = 1.32, *P* = .002; rs1329057, OR = 1.30, *P* = .003; rs5030717, OR = 1.34, *P* = .002). However, in three other cohorts (two in the United States and one in the United Kingdom), the three SNPs failed to show an association with the risk for OM. Finally, a variant, Asp299Gly (rs4986790) in *TLR4*, was found to be related to the colonization of *M. catarrhalis* in the upper respiratory tract of children (43%) in comparison with the *TLR4* wild type (9%) (RR = 4.91, P = .0001) (Hafren et al., 2015).

Lastly, another large-scale candidate gene study investigated the role of *FUT2*, a gene whose protein product regulates the expression of red blood cell Lewis and ABO antigens and that had previously been associated with increased risk of recurrent urinary tract infections, in familial OM (Santos-Cortez et al., 2018). For this study, families with children affected by OM were recruited. The participants were recruited from various ethnic backgrounds, including 137 individuals of indigenous Filipino descent (Santos-Cortez et al., 2015), 257 trios of mother, father, and child from Texas, 76 individuals from Colorado, 140 families from a previous family cohort from the University of Minnesota (Sale et al., 2011), 105 families from Helsinki University Hospital (Hafren et al., 2012), and 19 families from the southern Punjab province of Pakistan (Santos-Cortez et al., 2018). This study suggests an association of the c.412C > T variant of *FUT2* with chronic or recurrent OM in European-American children and family trios in the United States. Additionally, an association between the c.461G > A variant and transmission in family trios in the United States as well as shifts in the microbiota composition in the ME were observed. Lastly, they reported an association of the c.604C > T variant with OM in the Filipino data set, though 17 individuals did not show shifts in ME microbiota composition due to this variant. The authors proposed that *FUT2* variants confer higher risk for OM by modification of the microbiome in the ME. The authors also tested the expression and localization of *Fut2* and found that there is a transient increase in expression in the ME of mice inoculated with *H. influenzae* (Santos-Cortez et al., 2018). This study’s extensive array of results seems to suggest that different genetic variants and environmental factors influence susceptibility to OM (Santos-Cortez et al., 2018).

These candidate gene association studies have moved the field forward by suggesting an association between OM susceptibility and these various genetic variants, but unfortunately the statistical power of these studies remains quite low. Up to now, most existing association studies in OM have been poorly phenotyped and used small sample sizes (Bhutta, 2013), which significantly reduces the statistical power, decreasing thus the probability of replication. The latter can also be due to variation in study design, particularly if phenotypes are poorly matched. For example, there is some evidence suggesting that chronic OM should be evaluated and analyzed as distinct from acute OM. Yet, many studies on genetic risk of chronic OM have used candidate loci involved in innate or acute inflammatory pathways and included cases of both recurrent acute and chronic OM. Furthermore, epidemiological studies have demonstrated that AOM is a risk factor for ME effusion, although many children with COME have no preceding history of AOM (Bhutta, 2014). Also, mouse transcript studies have shown that genes upregulated in the initial phase of inflammation are different to those found as inflammation resolves, suggesting that there is a molecular transition from acute to chronic inflammation (Hernandez et al., 2015). Other reasons for failure of replication may be that the genetic structure might be disparate (Lao et al, 2008), or that different pathogenic mechanisms exist in different populations.

### Genome-Wide Association Studies

Since very few genes that contribute to the development of OM had been identified, genome-wide association studies (GWAS) have been used to decipher the genetic etiology of the disease by assessing whether any genetic variants throughout the entire genome associate with cases of OM (**Table 3**). A study recruited 416 cases (at least 3 episodes of AOM by the age of 3) and 1,075 controls from the Western Australian Pregnancy Cohort (Raine) Study, a longitudinal cohort study in Western Australia of 2,868 children whose mothers were recruited in early pregnancy from 1989 to 1991 (Rye et al., 2012). Despite a predominantly Caucasian cohort, it was reported that principle component analysis showed evidence for population stratification, or differences in allelic frequencies between subpopulations. The data set was corrected to account for this population substructure by adjusting for two principal components. Genome-wide association analysis of the corrected data set identified various SNPs showing association, the strongest of which were at *CAPN14* and *GALNT14* on chromosome 2p23.1. When the data set was tested without accounting for the population stratification, analysis did not reveal one significant genetic variant. Replication of the results was also attempted in a cohort of 793 affected individuals (55% with recurrent AOM and 45% with COM with effusion) from the Western Australian Family Study of OM. Twenty SNPs within seven candidate genes, including *CAPN14* and *GALNT14*, were genotyped, but no significant variants were identified. It was proposed that the lack of replication might be due to phenotypic and sample size differences in the populations (Rye et al., 2012). Another study attempted to replicate the results from the Western Australian Pregnancy Cohort (Raine) Study. Twenty-six autosomal SNPs from the Raine Study were analyzed for association in the sample of families with children affected by chronic OM with effusion and recurrent OM from the Daly et al. (2004), study discussed previously (Allen et al., 2014). No significant association was found with any of the 26 SNPs tested; the p-values ranged from 0.03 to 0.93, never crossing the significance threshold of p = 0.001 (Allen et al., 2014).

**Table 3:** List of genes implicated in otitis media from genomic association studies.

Gene	Allele or variant	Chromosome	Odds ratio, LOD score, or p-value	Case definition	Reference
*FBXO11*	A allele at rs10490302	2p16.3	OR 1.17	COME: symptomatic effusion for at least 3 months and effusion at operation	Bhutta et al., 2017
	G allele at rs2537742		OR 1.16		
*TGIF1*	T allele at rs881835	18p11.31	OR 1.39		
	G allele at rs1962914		OR 1.58		
*FUT2*	c.412C > T at rs1800022	19q13.33	p = 0.01*	Study assessed multiple different cohorts—case definition slightly different for each cohort	Santos-Cortez et al., 2018
	c.461G > A at rs601338		p = 0.01*		
	c.604C > T at rs1800028		LOD 4.0		
*CAPN14*	rs6755194	2p23.1	OR 1.90	Clinical exam within 3 years of life indicated presence of inflamed/retracted/scarred tympanic membrane, middle ear effusion, or tympanostomy tube OR parents reported 3 or more episodes of AOM within first 3 years of life	Rye et al., 2012
*GALNT14*	rs1862981		OR 1.60		
*KIF7*	rs1110060	15q26.1	p = 9.1 x 10^−7 a^p = 0.072 ^b^	Insertion of tympanostomy tube at least once for recurrent/persistent OM	Allen et al., 2013
*CDCA7 and SP3^d^*	rs10497394	2q31.1	p = 2.9 x 10^−5 a^p = 4.7 x 10^−5b^		
*FNDC1*	rs2932989	6q25.3	p = 4.36 x 10^−8 a^p = 2.15 x 10^−9c^	CHOP cohort: AOM defined using ICD-9 codes Generation R Study cohort: survey data on OM, otorrhea, fever with earache, and/or use of ear drops per subscription	Van Ingen et al., 2016

*Transmission disequilibrium test. ^a^Initial data. ^b^Replication data. ^c^Combined meta-analysis of initial and replication data. ^d^These genes border the corresponding marker, rs10497394.

A second GWAS study attempted to elucidate potential genes by genotyping 229 controls and 373 children who received tympanostomy tube surgery to treat COM with effusion or recurrent OM (Allen et al., 2013). Analysis of 324,748 SNPs revealed various SNPs on a number of chromosomes that showed suggestive or strong association with OM. The strongest of these variants was rs1110060 on chromosome 15 (p = 9.1 X 10^−7^). Replication of the findings from this genome-wide association analysis was attempted in a cohort of 652 controls and 932 affected children who also received tympanostomy tube insertion for chronic or recurrent OM (Allen et al., 2013). Of the 53 tested for replication genotyping, 4 significant SNPs (p < 0.10) were identified. One of these 4 SNPs was rs1110060 on chromosome 15 (p = 0.072), the most significant result from the initial study, but rs10497394 on chromosome 2 was the most significant replication (initial study: p = 2.9 X 10^−5^; replication study: p = 4.7 X 10^−5^) (Allen et al., 2013). The significant SNP variants identified in this study have been added to the list of candidate genes, such as *KIF7* (in rs1110060) and *CDCA7* and *SP3* (which both border rs10497394), that require further research (Allen et al., 2013).

Another study performed a meta-analysis of two cohorts consisting of 825 cases/7,936 controls and 1,219 cases/1,067 controls from the Children’s Hospital of Philadelphia (CHOP) and the Generation R Study (GenR) at Erasmus University Medical Center, the Netherlands, respectively (Van Ingen et al., 2016). The cases were defined as individuals with AOM prior to 3 years old. The GWAS suggested a significant association between the variant rs2932989 on chromosome 6q25.3 and AOM (p = 2.15 X 10^−9^). These findings were then replicated in an independent cohort of 293 cases of AOM and 1,719 controls recruited from the Children’s Hospital of Philadelphia. This SNP lies in an intergenic region near the *FNDC1* locus, so expression and methylation tests were performed to determine whether this gene could be associated with these findings. FNDC1 expression was found in multiple tissue types in mice and humans, in addition to mice ME (Van Ingen et al., 2016). The expression of *Fndc1* was also upregulated in mouse ME after treatment with lipopolysaccharide, which induces an inflammatory response and stimulates TGF-beta, TNF-alpha, and IL-1 signaling (Van Ingen et al., 2016). Lastly, the increase in *Fndc1* expression may be explained by experiments that show lower levels of methylation of human *FNDC1* in the SNP of interest (Van Ingen et al., 2016).

Although these GWAS present promising results of associations between specific genetic markers and OM, GWAS also struggles with the dichotomy of high false positive rates and lower than desired power. Commonly in GWAS, the false positive rate is maintained at 5%. As a result, though, the study loses power because the p-value threshold becomes very small in order to be considered significant (Tam et al., 2019). Since the p-value is so low, there can be a number of SNPs that have associations with the trait that do not get detected because their p-value does not cross the very strict threshold. Even though there are replicable associations between some genetic variants and OM, there still remains the issue of causality. A GWAS on its own cannot explain the role of the genetic variants on the pathophysiology of OM, so studies must be carried out to further elaborate the potential mechanistic involvement of these genes.

### Genetics and the Microbiome

The composition and diversity of the microbiome, as well as the exogenous and endogenous factors that affect it, is notoriously difficult to study. However, high-throughput sequencing analysis techniques such as 16S rRNA and metagenomics (MGS) have contributed significantly to the field, allowing a more detailed study of microbiome composition, specific genetic loci, and disease progression (Turnbaugh et al., 2009; Yatsunenko et al., 2012; Kurilshikov et al., 2017; Awany et al., 2019). A more comprehensive study is valuable in light of the growing understanding of how the microbiome can either prevent or foster infectious, metabolic, inflammatory, atopic, and neurological disease through predominantly immuno-modulatory processes (Tremlett et al., 2017; Aydin et al., 2018; Imhann et al., 2018; Lee et al., 2018; Liang et al., 2018). Of note, these advancements have been facilitated by the development of microbial genome-wide association studies (mGWAS) techniques to probe the genome for loci directly influencing bacterial populations. mGWAS studies have already identified hundreds of microbial quantitative trait loci (mbQTLs) associated with alpha-diversity, beta-diversity, and abundance of particular taxonomic groups and have mapped them to polymorphisms related to mucosal immunity and metabolic processes (Goodrich et al., 2014; Bonder et al., 2016; Turpin et al., 2016; Wang et al., 2016; Hall et al., 2017; Kurilshikov et al., 2017). For example, innate immune factors such as C-type lectins, toll-like receptor (TLR) regulators including *Irak2*, and NOD-like receptors (*NOD1/NOD2*) have all been shown to affect microbial environment, while genes such as *FUT2, LCT, CARD9* have linked the host and microbiota metabolism, all of which carry implications for disease susceptibility, progression, and treatment (Blekhman et al., 2015; Lamas et al., 2016; Kurilshikov et al., 2017).

In an attempt to extricate the environmental impact from that of a host’s genome, a study reviewed mGWAS studies and methodologies and reports that mGWAS’s earliest use helped identify 83 microbiome-loci associations, with follow-up studies especially supporting the discovered link between the *LCT* lactase gene and *Bifidobacterium* (Blekhman et al., 2015; Weissbrod et al., 2018). Meanwhile, other studies demonstrated that 10.43% of microbiome variability (beta-diversity) between individuals can be attributed to 42 genetic loci, and that microbial diversity is particularly affected by the *VDR* (vitamin D receptor) gene (Wang et al., 2016; Hall et al., 2017). However, the majority of these existing mGWAS studies analyze gut microbiota, thereby limiting the direct application to the OM field. One of the only mGWAS studies exploring the composition of the nasal vestibule and NP microbiome was performed using 16S rRNA methods to compare a small, 144-person cohort with the alpha diversity, beta diversity, and relative abundance of bacterial taxa (Igartua et al., 2017). The study identified 37 significant associations (mbQTLs) out of 148,653 genetic variants (q < 0.05), the most significant of which linked *Dermacoccus* and the variant rs117042385 located upstream of terminal differentiation induced non-coding RNA, a non-coding RNA binding to the mRNA of *PGLYRP3* encoding a known antimicrobial protein important for epidermal differentiation (Igartua et al., 2017). Importantly, the study also found a significant association between shared genetics and similar microbiome composition even after controlling for a shared household environment. Igartua et al. therefore was able to establish an association between genes, microbial populations in the upper airways, and innate mucosal immunity, thereby encouraging the further use of this new technology to the study of OM and other upper respiratory tract infections.

It is important to note some limitations of these new contributions. The conflicting results from analyses of the same data sets have been reported, though this has been ascribed to differences in statistical methods and significance cutoffs. Also, much like the limitations introduced by GWAS studies, associations identified by mGWAS are often weak at best, spread out across the genome, and therefore impose a comparatively insignificant effect. Finally, the ever-changing nature of the microbiome—a bacterial population susceptible to both short- and long-term changes influenced by diet, transient infections, and a slew of other behavioral variations of the host—complicates reproducibility of data. Therefore, carefully designed studies are needed in both the OM and the gastrointestinal fields in order to clarify links between a fixed, heritable genome to a variable as dynamic as the microbiome.

### Otitis Media and the Microbiome

The microbiome has been implicated in the pathogenesis of OM, primarily in its role as an immunomodulator and as a competitor against invading bacteria seeking both nutrients and space. A number of studies have determined alteration in microbiome profile in OM prone individuals (**Table 4**). As mentioned before, the microbiome may carefully modulate host inflammatory processes through intricate cross talk between immune cells and the resident microbiota (Sokolowska et al., 2018). Effective communication between host commensals and host immune cells is essential for both dampening inflammatory cascades when not required and promoting immune responses against invading pathogens threaten stability. The composition and integrity of the microbiome is therefore a key factor in OM pathogenesis, and next-generation sequencing technologies have allowed us to better study gene-environment interactions at this microscopic level. Herein we explore the association between both the NP and ME microbiota and their relationship with invasive, OM-causing pathogens.

**Table 4:** A summary of studies determining microbiome of upper respiratory tract in individuals susceptible to otitis media.

Sample type	Age of subjects	Sample size	Results	References
MEF, NP swab, adenoid swab	3–10 years	11 indigenous children with OME	*- Alloiococcus, Haemophilus, Streptococcus, Moraxella*	Jervis-Bardy et al., 2015
Nasal aspirates	2–12 months	234 infants	- *Moraxella, Streptococcus, Haemophilus*	Teo et al., 2015
MEF, adenoid swab	1–12 years	23 children with COME, 10 children without COME with respiratory surgery	- *Alloiococcus* inversely related to *Haemophilus* - Abundance of *Alloiococcus otitidis* and *Staphylococcus* in MEF	Chan et al., 2016
ME and mastoid swab	6 months–87 years	46 children/adults with COM	*- Haemophilus, Staphylococcus, Alloiococcus*	Neeff et al., 2016
MEF and external auditory canal lavage	1–14 years	18 children with OME	- Predominance of *A. otitidis* and *Haemophilus* in MEF - Abundance of *A. otitidis* and *Staphylococcus* in ear canal	Chan et al., 2017
MEF	5 months–2.5 years	79 children with AOM	- Predominance of *S. pneumoniae*, *Haemophilus influenza*, and *Moraxella catarrhalis* - *A. otitidis, Turicella otitidis*, and *Staphylococcus auricularis*	Sillanpää et al., 2017
NP swab	<12 months	65 children with AOM; 74 children without AOM	- *Staphylococcus* and *Sphingobium* associated with decreased risk of AOM development - Infants having AOM showed reduction in numbers of *Corynebacterium*	Chonmaitree et al., 2017
MEF	3 months–14.6 years	55 children with COME	*- Haemophilus, Moraxella, Turicella*	Krueger et al., 2017
MEF and NP swab	<12 years	9 children with gastroesophageal reflux-associated OM; 21 children with OM only	- *Alloiococcus* and *Turicella* in MEF - *Haemophilus* and *Streptococcus* detected in MEF if also present in NP -No difference between reflux and non-reflux microbiota	Boers et al., 2018
MEF and ME aspirates, ear canal swab, nasal swab	0–60 months	93 children with rAOM and 103 healthy controls	*- Haemophilus, Turicella, Alloiococcus, Staphylococcus*	Lappan et al., 2018
MEF (otorrhea) and NP swab	<5 years	94 children with AOM+ grommets	- MEF had less microbial diversity than nasopharynx - There was an abundance of *Corynebacterium* and *Dolosigranulum*	Man et al, 2019a
NP adenoid swabs, palatine tonsillar swabs	15–65 years	14 adults with tonsil/adenoid hyperplasia; 14 adults with secretory OM	- *S. pneumoniae*, *H. influenzae*, and *M. catarrhalis* exclusively in adenoids	Fagö-Olsen et al., 2019
ME, adenoid, and tonsil swab	2–10 years	10 children with OME	*- Fusobacterium, Haemophilus, Neisseria, Porphyromonas*	Johnston et al., 2019

### Microbiome of the Nasopharynx

Microbiome ecosystems are highly specific to their immediate environment, and their composition can vary widely depending on their anatomic location within the host. For this reason, studies have repeatedly uncovered distinct resident microbiota compositions in the gut, upper respiratory tract, NP, and both the middle as well as the inner ear (Cho and Blaser, 2012). However, AOM research often cites the first step of pathogenesis as bacterial colonization and/or viral disturbance of the NP, from which the disease eventually progresses through the Eustachian tube to the ear. Animal studies have also demonstrated this time course, with initial inoculation of bacteria in the NP followed by eventual migration to the ME, where they may persist for days, weeks, and even months (Cripps and Kyd, 2003; Coticchia et al., 2013; Schilder et al., 2016; Dewan et al., 2019). Thus, analysis of both the ME and NP microbiota is essential for obtaining a comprehensive understanding of the host-pathogen interactions leading to OM development. However, obtaining nasopharyngeal swab samples is often easier, cheaper, and less invasive than samples from the ME. Therefore, the nasopharyngeal microbiome has been more extensively studied than the ME microbiome.

The various morphologies and presentations of OM disease [AOM, recurrent AOM (rAOM), CSOM, OME, and/or COM] is about as varied and dynamic as the bacterial, viral, and fungal microorganisms that contribute to OM pathogenesis. However, the research has well-established the link between rAOM and the major otopathogens *S. pneumoniae*, non-typeable *Haemophilus influenza* (NTHi), and *M. catarrhalis*, and these species are consistently found at higher levels of the NP in studies comparing the microbiota of rAOM and COME cases *versus* healthy controls (Lappan et al., 2018; Walker et al., 2019). Yet the presence of these same organisms, albeit at lower levels, in the controls suggest their potential role as endogenous pathobionts even in the absence of disease. NTHi was found to be the most abundant of three otopathogens in the ME with 18.5% of ME reads attributable to this species, though NTHi was still more abundant in the NP than the ME of rAOM cases. A study sequencing ME fluids/rinses, ear canal swabs, and NP using 16S rRNA observed that *Neisseria* (10.9% RA) was associated with cases of AOM specifically, while *Gemella* (3.2% RA), *Porphyromonas* (3.6% RA), *Alloprevotella* (2.4% RA), and *Fusobacterium* (2.7% RA) were also present in cases but absent (<0.3% RA) in the NP of healthy controls (Lappan et al., 2018). Taken together, the unique presence of these bacteria in rAOM cases has demonstrated an increased level of microbiota diversity in cases compared to healthy controls.

This finding is somewhat controversial, as many other studies have instead shown increased diversity in healthy controls compared to OM cases, suggesting increased bacterial diversity as a protective factor against invading pathogenic bacteria. For example, one recent study compared the NP microbiota of 3 to 4-year-old patients undergoing tympanostomy for COME and found overall microbiota diversity to be decreased in cases compared to controls, as measured by both a lower Shannon diversity index as well as cluster analyses showing an increased number of profiles dominated by one spp. in the case compared to the control group (Walker et al., 2019). However, the group’s analysis still found greater levels of standard otopathogens in cases, as well as a greater level of expected commensal species such as a-hemolytic *Streptococci* and *Lactococci* in controls (Walker et al., 2019). The discrepancy in microbial diversity results may be attributable to the differences in disease processes between acute COM (aCOM) and COME, as the pathogenesis of COME distinctly occurs when bacteria in the biofilm state trigger and maintain a mucoid or serous inflammatory effusion.

Nevertheless, diversity analyses warrant future study, as only about half of all existing studies comparing microbiome diversity between AOM cases *versus* control samples have also found diversity to be higher in healthy controls. Currently, alterations in microbiome diversity are generally attributed to antibiotic use. Studies have explored how microbiota populations are near-permanently altered following the administration and completion of antibiotic regimens, with single opportunistic pathogens initially dominating but new pathogens establishing with time (Kaur et al., 2013). Of note, all current data in the literature are acquired from cross-sectional cohort studies involving diseased and healthy populations; therefore, the temporality of the association cannot be empirically established. Other than chronic antibiotic use, alternative hypotheses to explain the link between increased microbial diversity and increased incidence of disease include the role of synergism in polymicrobial etiologies of OM. Animal-model studies utilizing bacterial and viral co-infections have been shown to significantly increase the incidence of bacterial OM. For example, recent studies demonstrate that the presence of different bacteria not only affects the rates and burden of nasopharyngeal colonization but can also enhance the incidence and severity of OM (Krishnamurthy et al., 2009). Other studies have also noted that *M. catarrhalis* is more likely to be observed within mixed bacterial observations than isolated alone, and that this polymicrobial environment can affect the incidence of *S. pneumoniae* OM (Krishnamurthy et al., 2009; Pichichero, 2009).

As OM disease more commonly affects pediatric populations, studies aiming to discern the distinct microbiome of children have helped uncover differences in the microbiota of healthy children *versus* those suffering from respiratory disease. For example, a prospective birth cohort study featuring 1,616 samples from healthy children and 1,423 samples from children with respiratory disease highlighted microbiome profile groups dominated by *Haemophilus*, *Streptococcus*, *Moraxella*, *Staphylococcus*, *Corynebacterium*, and *Alloiococcus* in 91% of samples (Lang et al., 2019). Importantly, the study noted increased abundances of *Haemophilus* (OR 3.9, p58.4x10−16), *Streptococcus* (OR 3.8, p58.9x10−18), and *Moraxella* (OR 2.1, p53.4x10−12) within the context of disease, while *Staphylococcus*, *Corynebacterium*, and *Alloiococcus* profiles were inversely correlated with disease (OR 0.22/p = 52.5x10−4, OR 0.49/p = 53.8x10−9, and OR 0.25/p = 53.7x10−17, respectively). Meanwhile, an elegant case-control study performed in the Netherlands found similar microbiome compositions within the lower respiratory tract and the NP of children, while also demonstrating that microbial profiles dominated by *H. influenzae/haemolyticus* and *S. pneumoniae* were associated with disease (Man et al., 2019b). The study also identified the importance of a combined classification model (bacterial microbiome, viral microbiome, and external factors such as antibiotic use and breastfeeding) in reliably predicting clinical disease, thereby stressing the multifactorial nature of disease (Man et al., 2019b).

### *Dolosigranulum and Corynebacterium*: The Discovery and Role of Protective Bacteria

While the comparison of NP microbiota generally highlights the unique presence of bacterial species in AOM cases rather than controls, two species of bacteria have instead emerged in increased abundance within the NP microbiome of healthy volunteers. The increased relative abundance of *Dolosigranulum* (16.34% case RA/1.86% control RA) and *Corynebacterium* (13.34% case RA/1.63% control RA) suggests the potential protective role of these bacteria against competing pathological bacteria seeking space and nutrients within the respiratory tract ecosystem (Lappan et al., 2018). The two species were found to correlate together, thereby suggesting a symbiotic relationship potentially mediated by *Dolosigranulum’*s production of lactic acid into the environment. These findings are supported by previous reports of *Dolosigranulum* and *Corynebacterium* predominating the NP of both healthy adults and children, their association with a decreased risk of OM/AOM diagnosis or pneumococcal colonization, and a shorter duration of otorrhea (Laufer et al., 2011; Pettigrew et al., 2012; Bomar et al., 2016; Chonmaitree et al., 2017; Man et al., 2019a). Interestingly, other studies have linked increased *Dolosigranulum* and *Corynebacterium* levels with both breastfeeding and vaginal (compared to *Cesarean*) deliveries, thereby suggesting a mechanism to explain the reported link between breastfeeding and OM resistance (Biesbroek et al., 2014; Biesbroek et al., 2014b).

As described previously, however, the diminished presence of these species in rAOM case cohorts may instead be attributed to rAOM patients’ heightened exposure to long-term antibiotics, and studies have indeed found *Dolosigranulum* and *Corynebacterium* relatively depleted in patients with recent history of children using antibiotics (Pettigrew et al., 2012). Antibiotic treatments not only disrupt the host microbiome by temporarily reducing certain bacterial populations, but are also capable of inducing long-term dysbiosis, leaving hosts susceptible to infection as well as atopic and inflammatory diseases (Francino, 2015). Despite this, some experiments have attempted to identify a causal link between the abundance of these two species and the health of the NP. One study uncovered a mechanism in which *Corynebacterium* species *Corynebacterium accolens* was able to utilize triacylglycerols found on the human skin to assemble free fatty acids that inhibited growth of S. pneumoniae (Bomar et al., 2016). Another report found *Corynebacteria* to eradicate *S. aureus* in healthy nasopharynges of adults, and yet another demonstrated how the introduction of *Corynebacteria* helped mice resist both respiratory syncytial virus and secondary pneumococcal infection, presumably through host immune modulation (Uehara et al., 2000; Kanmani et al., 2017). Preliminary *in vitro* experiments show multiple strains of *Corynebacteria* inhibiting growth of *M. catarrhalis*, and though the mechanism is still unclear, it appears more a result of resource competition than particular bactericidal activity (Lappan, 2019). The potential of these two species as protection against OM-causing bacteria certainly warrants further exploration, especially in light of the potential to develop novel probiotic therapies that would reduce antibiotic use. Other species demonstrating a potential clinical benefit, such as *Haemophilus haemolyticus* and *Streptococcus salivarius*, also present the opportunity for further therapeutic development (Marchisio et al., 2015; Pickering et al., 2016). Future research should incorporate *in vivo* models to incorporate relevant host immune factors, address the impact of environmental factors that modulate host-pathogen interaction (pH, lactic acid, etc.), as well as clinical studies that control for past antibiotic exposure.

### Middle Ear Microbiome

The use of nasopharyngeal washes and/or swabs as a less invasive methodical approach for the analysis of host microbiota has produced an abundance of NP microbiome analyses. Meanwhile, studies involving the extraction of ME samples are rare. One study attempting to address this discrepancy by obtaining ME saline washes and ME fluids of children undergoing grommet insertion for rAOM (Lappan et al., 2018). Using 16S rRNA sequencing methods, researchers identified *Alloiococcus otitidis*, *Turicella otitidis*, and *Staphylococcus* spp. as uniquely present in the ME of patients undergoing grommet insertion for severe rAOM diagnoses. The absence of these species in nasopharyngeal swab samples also obtained from the same patients suggests these species’ role as normal auricle flora and/or as standard ME pathogens in cases of OM (Lappan et al., 2018). The standard otopathogens *S. pneumoniae*, NTHi, and *M. catarrhalis* were found to be present in the ME of rAOM cases, albeit at lower levels than in the NP, and also in lower levels than the standard ME pathogens previously described. However, their presence in the ME suggests bacterial migratory patterns originating in the oropharynx and extending through the Eustachian tube and into the ME, which is consistent with known OM pathogenesis. Finally, ME diversity was found to be lower than NP diversity in case samples, further establishing the NP’s role as a robust reservoir for microorganisms in both the presence and absence of disease (Lappan et al., 2018).

#### Genetics, The Microbiome, and Otitis Media

As previously described, a healthy microbiome is dependent on effective communication between host commensals and host immune systems, with microbiome composition and diversity in part determined by the host genome. Therefore, genetic defects affecting key biomolecular messengers/signals within this network of crosstalk can have profound effects on the integrity of this balance and therefore the microbiome itself. Within the specific context of OM pathogenesis, researchers have discovered a candidate gene that seems to shape micro-environments favoring particular groups of OM pathobionts. A rare duplication variant of *A2ML1* (α2-macroglobulin-like 1), a gene encoding a middle-ear specific protease inhibitor, has been identified to confer an increased susceptibility to OM (Santos-Cortez et al., 2015). The study incorporated samples from a 250-member intermarried indigenous Filipino community with a 49.6% prevalence of OM, which ultimately identified the *A2ML1* duplication c.2478_2485dupGGCTAAAT (p.Ser829Trpfs*9) as co-segregating with OM with a logarithm of odds (LOD) of 7.5. The study also compared these findings with 123 otitis-prone and 118 non-otitis prone children from the University of Texas Medical Branch (UTMB). Within the Texas cohort, 3 of the 123 otitis-prone children were found to have the same A2ML1 variant, all of whom reported early-onset severe OM that ultimately required tympanostomy tube insertion surgeries.

While this initial study identified the candidate gene and therefore suggested the genetic link to OM susceptibility, follow-up studies uncovered the host microbiome composition as an essential mediator of the association. Using 16S rRNA gene sequencing for microbiome analysis of outer and ME, samples from carriers of the *A2ML1* variant were found to have higher levels of *Fusobacterium, Porphyromonas, Peptostreptococcus, Parvimonas*, and *Bacteroides* compared to wild-type COM cases, and variant carriers also demonstrated an increased alpha-diversity *via* mean Shannon diversity index (Santos-Cortez et al., 2016). Meanwhile, microbiota from wild-type cases were more likely to feature well-known otopathogens such as *Alloiococcus*, *Staphylococcus, Proteus*, and *Haemophilus*. It is important to note that study limitations were characterized by issues of statistical significance due to small sample sizes, as the study cohort included only 16 indigenous Filipino patients, 11 of whom carry the *A2ML1* variant. However, these studies are the first of their kind in that they specifically apply the genome-microbiome interaction to OM disease susceptibility, offering exciting new avenues for further exploration in our field especially.

## Conclusion and Future Directions

Genetic predisposition to OM has been elucidated through twin studies, linkage association studies, candidate gene approaches, and genome-wide association studies, but further research is necessary to precisely understand the genetic etiology and determine which genes are at play. Through these studies, a number of genes and SNPs have been implicated, including *FBXO11, TGIF1, FUT2, CAPN14, GALNT14, KIF7, CDCA7, SP3*, and *FNDC1*, yet replication of these results has been unsuccessful. One major issue with replication of genetic findings is that it is incredibly difficult to find cohorts with the exact same clinical phenotype for OM. Further, some cohorts focus on healthy *versus* AOM, others focus on acute OM *vs.* recurrent OM/COME. In addition to these studies, a growing body of research incorporating bioinformatical analysis of host microbiota is beginning to identify how these particular genes influence OM susceptibility through mediation of the host microbiota, such as the *A2ML1* variant’s association with *Fusobacterium* and *Bacteroides* anaerobic bacteria within the NP of chronic AOM patients. Meanwhile, studies are comparing resident host microbiota of healthy controls *versus* COM cases within both the NP and the ME/ear canal. While data from these studies reinforce conclusions regarding *S. pneumoniae*, NTHi, and *M. catarrhalis* as primary otopathogens, they also found these three species at higher levels in the NP than the ME, which was instead predominantly colonized with *Staphylococcus* spp., *A. otitidis*, and *T. otitidis*. However, these studies demonstrate the highest level of bacterial diversity in the NP of OM cases when compared to NP and ME bacterial populations within both case and control patients. These findings challenge a series of studies that have found higher bacterial diversity to be associated with healthy microbiota compared to COME microbiota, though the controversy reinforces the need to establish temporality in future studies due to antibiotic use as a potential confounding variable. These studies also have identified higher levels of *Dolosigranulum* and *Corynebacterium* in healthy control NP microbiota, thereby suggesting a potential protective effect that may be harnessed in the form of novel probiotic therapies. This hypothesis must be tested within the context of longitudinal studies, as *Dolosigranulum* and *Corynebacterium* may simply be more susceptible to long-term antibiotic use more prevalent in the aCOM case group. However, as studies highlighting the gene-microbiome interactions are especially new and offer the greatest potential in the coming years, we anticipate the expansion of new insights that may be applicable to the development of novel probiotic therapies for OM.

## Author Contributions

All the authors listed contributed intellectually in writing of the manuscript and approved the final version of the manuscript for publication.

## Funding

Dr. Liu’s Laboratory is supported by R01 DC005575, R01DC017264, T32 DC015995, and R01 DC012115 grants from the National Institutes of Health/National Institute on Deafness and Other Communication Disorders.

## Conflict of Interest

The authors declare that the research was conducted in the absence of any commercial or financial relationships that could be construed as a potential conflict of interest.

## References

[B1] Ahmed S. Shapiro N. L. Bhattacharyya N. (2014). Incremental health care utilization and costs for acute otitis media in children. Laryngoscope. 124 (1), 301–305. 10.1002/lary.24190 23649905

[B2] Allen E. K. Chen W. M. Weeks D. E. Chen F. Hou X. Mattos J. L. (2013). A genome-wide association study of chronic otitis media with effusion and recurrent otitis media identifies a novel susceptibility locus on chromosome 2. J. Assoc. Res. Otolaryngol. 14 (6), 791–800. 10.1007/s10162-013-0411-2 23974705 PMC3825021

[B3] Allen E. K. Manichaikul A. Chen W. M. Rich S. S. Daly K. A. Sale M. M. (2014). Evaluation of replication of variants associated with genetic risk of otitis media. PloS One 9 (8), e104212. 10.1371/journal.pone.0104212 25089819 PMC4121324

[B4] Awany D. Allali I. Dalvie S. Hemmings S. Mwaikono K. S. Thomford N. E. (2019). Host and microbiome genome-wide association studies: current state and challenges. Front. Genet. 9, 637. 10.3389/fgene.2018.00637 30723493 PMC6349833

[B5] Aydin Ö. Nieuwdorp M. Gerdes V. (2018). The gut microbiome as a target for the treatment of type 2 diabetes. Curr. Diabetes Rep. 18 (8), 55. 10.1007/s11892-018-1020-6 PMC6013535 29931613

[B6] Bardach A. Ciapponi A. Garcia-Marti S. Glujovsky D. Mazzoni A. Fayad A. (2011). Epidemiology of acute otitis media in children of Latin America and the Caribbean: a systematic review and meta-analysis. Int. J. Pediatr. Otorhinolaryngol 75, 1062–1070. 10.1016/j.ijporl.2011.05.014 21665297

[B7] Bhutta M. F. Lambie J. Hobson L. Goel A. Hafrén L. Einarsdottir E. (2017). A mouse-to-man candidate gene study identifies association of chronic otitis media with the loci TGIF1 and FBXO11. Sci. Rep. 7 (1), 12496. 10.1038/s41598-017-12784-8 28970529 PMC5624881

[B8] Bhutta M. F. Cheeseman M. T. Herault Y. Yu Y. E. Brown S. D. (2013). Surveying the Down syndrome mouse model resource identifies critical regions responsible for chronic otitis media. Mamm. Genome 24, 439–45. 10.1007/s00335-013-9475-x PMC3843744 24068166

[B9] Bhutta M. F. (2014). Epidemiology and pathogenesis of otitis media: construction of a phenotype landscape. Audiol. Neurotol. 19 (3), 210–223. 10.1159/000358549 24819621

[B10] Biesbroek G. Bosch A. Wang X. Keijser B. Veenhoven R. Sanders E. (2014a). The impact of breastfeeding on nasopharyngeal microbial communities in infants. Am. J. Respir. Crit. Care Med. 190 (3), 298–308. 10.1164/rccm.201401-0073OC 24921688

[B11] Biesbroek G. Tsivtsivadze E. Sanders E. Montijn R. Veenhoven R. Keijser B. (2014b). Early respiratory microbiota composition determines bacterial succession patterns and respiratory health in Children. Am. J. Respir. Crit. Care Med. 190 (11), 1283–1292. 10.1164/rccm.201407-1240OC 25329446

[B12] Blekhman R. Goodrich J. K. Huang K. Sun Q. Bukowski R. Bell J. T. (2015). Host genetic variation impacts microbiome composition across human body sites. Genome Biol. 16 (1), 191. 10.1186/s13059-015-0759-1 26374288 PMC4570153

[B13] Boers S. A. de Zeeuw M. Jansen R. van der Schroeff M. P. van Rossum A. M. C. Hays J. P. (2018). Characterization of the nasopharyngeal and middle ear microbiota in gastroesophageal reflux-prone versus gastroesophageal reflux non-prone children. Eur. J. Clin. Microbiol. Infect. Dis. 37 (5), 851–857. 10.1007/s10096-017-3178-2 29404836 PMC5916997

[B14] Bomar L. Brugger S. Yost B. Davies S. Lemon K. (2016). Corynebacterium accolens releases antipneumococcal free fatty acids from human nostril and skin surface triacylglycerols. mBio. 7 (1), e01725–e01715. 10.1128/mBio.01725-15 26733066 PMC4725001

[B15] Bonder M. J. Kurilshikov A. Tigchelaar E. F. Mujagic Z. Imhann F. Vich A. (2016). The effect of host genetics on the gut microbiome. Nat. Genet. 48 (11), 1407–1412. 10.1038/ng.3663 27694959

[B16] Bondy J. Berman S. Glazner J. Lezotte D. (2000). Direct expenditures related to otitis media diagnoses: extrapolations from a pediatric Medicaid cohort. Pediatrics. 105 (6), E72. 10.1542/peds.105.6.e72 10835085

[B17] Broides A. Dagan R. Greenberg D. Givon-Lavi N. Leibovitz E. (2009). Acute otitis media caused by Moraxella catarrhalis: epidemiologic and clinical characteristics. Clin. Infect. Dis. 49 (11), 1641–1647. 10.1086/647933 19886799

[B18] Caruso R. Mathes T. Martens E. C. Kamada N. Nusrat A. Inohara N. (2019). A specific gene-microbe interaction drives the development of Crohn’s disease–like colitis in mice. Sci. Immunol. 4 (34),eaaw4341. 10.1126/sciimmunol.aaw4341 31004013 PMC8882361

[B19] Casey J. R. Adlowitz D. G. Pichichero M. E. (2010). New patterns in the otopathogens causing acute otitis media six to eight years after introduction of pneumococcal conjugate vaccine. Pediatr. Infect. Dis. J. 29 (4), 304–309. 10.1097/INF.0b013e3181c1bc48 19935445 PMC3959886

[B20] Casey J. R. Kaur R. Friedel V. C. Pichichero M. E. (2013). Acute otitis media otopathogens during 2008 to 2010 in Rochester, New York. Pediatr. Infect. Dis. J. 32 (8), 805–809. 10.1097/INF.0b013e31828d9acc 23860479 PMC3755474

[B21] Casselbrant M. L. Mandel E. M. Fall P. A. Rockette H. E. Kurs-Lasky M. Bluestone C. D. (1999). The heritability of otitis media: a twin and triplet study. JAMA. 282 (22), 2125–2130. 10.1001/jama.282.22.2125 10591333

[B22] Casselbrant M. L. Mandel E. M. Rockette H. E. Kurs-Lasky M. Fall P. A. Bluestone C. D. (2004). The genetic component of middle ear disease in the first 5 years of life. Arch. Otolaryngol. Head Neck Surg. 130 (3), 273–278. 10.1001/archotol.130.3.273 15023832

[B23] Casselbrant M. L. Mandel E. M. Jung J. Ferrell R. E. Tekely K. Szatkiewicz J. P. (2009). Otitis media: a genome-wide linkage scan with evidence of susceptibility loci within the 17q12 and 10q22.3 regions. BMC Med. Genet. 10, 85. 10.1186/1471-2350-10-85 19728873 PMC2751750

[B24] Chan C. L. Wabnitz D. Bardy J. J. Bassiouni A. Wormald P. J. Vreugde S. (2016). The microbiome of otitis media with effusion. Laryngoscope 126 (12), 2844–2851. 10.1002/lary.26128 27335217

[B25] Chan C. L. Wabnitz D. Bassiouni A. Wormald P. Vreugde S. Psaltis A. J. (2017). Identification of the Bacterial Reservoirs for the Middle Ear Using Phylogenic Analysis. JAMA Otolaryngol. Head Neck Surg. 143 (2), 155–161. 10.1001/jamaoto.2016.3105 27812691

[B26] Chen W. M. Allen E. K. Mychaleckyj J. C. Chen F. Hou X. Rich S. S. (2011). Significant linkage at chromosome 19q for otitis media with effusion and/or recurrent otitis media (COME/ROM). BMC Med. Genet. 12, 124. 10.1186/1471-2350-12-124 21943191 PMC3191346

[B27] Chen J. Ingham N. Clare S. Raisen C. Vancollie V. E. Ismail O. (2013). Mcph1-deficient mice reveal a role for MCPH1 in otitis media. PloS One 8 (3), e58156. 10.1371/journal.pone.0058156 23516444 PMC3596415

[B28] Cho I. Blaser M. J. (2012). The human microbiome: at the interface of health and disease. Nat. Rev. Genet. 13 (4), 260–270. 10.1038/nrg3182 22411464 PMC3418802

[B29] Chonmaitree T. Revai K. Grady J. J. Clos A. Patel J. A. Nair S. (2008). Viral upper respiratory tract infection and otitis media complication in young children. Clin. Infect. Dis. 46 (6), 815–823. 10.1086/528685 18279042 PMC2744371

[B30] Chonmaitree T. Trujillo R. Jennings K. Alvarez-Fernandez P. Patel J. A. Loeffelholz M. J. (2016). Acute otitis media and other complications of viral respiratory infection. Pediatrics 137 (4), e20153555. 10.1542/peds.2015-3555 27020793 PMC4811317

[B31] Chonmaitree T. Jennings K. Golovko G. Khanipov K. Pimenova M. Patel J. (2017). Nasopharyngeal microbiota in infants and changes during viral upper respiratory tract infection and acute otitis media. PloS One 12 (7), e0180630. 10.1371/journal.pone.018063 28708872 PMC5510840

[B32] Coticchia J. M. Chen M. Sachdeva L. Mutchnick S. (2013). New paradigms in the pathogenesis of otitis media in children. Front. Pediatr. 1, 52. 10.3389/fped.2013.00052 24400296 PMC3874850

[B33] Cripps A. W. Kyd J. M. (2003). Bacterial otitis media: Current vaccine development strategies. Immunol. Cell Biol. 81 (1), 46–51. 10.1046/j.08018-9641.2002.01141.x 12534945

[B34] Daly K. A. Brown W. M. Segade F. Bowden D. W. Keats B. J. Lindgren B. R. (2004). Chronic and recurrent otitis media: a genome scan for susceptibility loci. Am. J. Hum. Genet. 75 (6), 988–997. 10.1086/426061 15514890 PMC1225283

[B35] Dewan K. K. Taylor-Mulneix D. L. Campos L. L. Skarlupka A. L. Wagner S. M. Ryman V. E. (2019). A model of chronic, transmissible otitis media in mice. PloS Pathog. 15 (4), e1007696. 10.1371/journal.ppat.1007696 30970038 PMC6476515

[B36] Fagö-Olsen H. Dines L. M. Sørensen C. H. Jensen A. (2019). The adenoids but not the palatine tonsils serve as a reservoir for bacteria associated with secretory otitis media in small children. mSystems 4 (1), e00169–e00118. 10.1128/mSystems.00169-18 PMC6372837 30801022

[B37] Ferreira M. A. (2004). Linkage analysis: principles and methods for the analysis of human quantitative traits. Twin Res. 7 (5), 513–530. 10.1375/twin.7.5.513 15527667

[B38] Francino M. P. (2015). Antibiotics and the human gut microbiome: dysbioses and accumulation of resistances. Front. Microbiol. 6, 1543. 10.3389/fmicb.2015.01543 26793178 PMC4709861

[B39] Gates G. A. (1996). Cost-effectiveness considerations in otitis media treatment. Otolaryngol. Head Neck Surg. 114 (4), 525–530. 10.1016/S0194-5998(96)70243-7 8643261

[B40] Gessner B. D. Gillingham M. B. Wood T. Koeller D. M. (2013). Association of a genetic variant of carnitine palmitoyltransferase 1A with infections in Alaska Native children. J. Pediatr. 163 (6), 1716–1721. 10.1016/j.jpeds.2013.07.010 23992672

[B41] Goodrich J. K. Waters J. L. Poole A. C. Sutter J. L. Koren O. Blekhman R. (2014). Human genetics shape the gut microbiome. Cell 159 (4), 789–799. 10.1016/j.cell.2014.09.053 25417156 PMC4255478

[B42] Hafren L. Kentala E. Jarvinen T. M. Leinonen E. Onkamo P. Kere J. (2012). Genetic background and the risk of otitis media. Int. J. Pediatr. Otorhinolaryngol. 76 (1), 41–44. 10.1016/j.ijporl.2011.09.026 22018929

[B43] Hafren L. Einarsdottir E. Kentala E. Hammaren-Malmi S. Bhutta M. F. MacArthur C. J. (2015). Predisposition to childhood otitis media and genetic polymorphisms within the Toll-like receptor 4 (TLR4) locus. PloS One 10 (7), e0132551. 10.1371/journal.pone.0132551 26177520 PMC4503307

[B44] Hall A. B. Tolonen A. C. Xavier R. J. (2017). Human genetic variation and gut microbiome disease. Nat. Rev. Genet. 18 (11), 690–699. 10.1038/nrg.2017.63 28824167

[B45] Han F. Yu H. Li P. Zhang J. Tian C. Li H. (2012). Mutation in Phex gene predisposes BALB/c-Phex(Hyp-Duk)/Y mice to otitis media. PloS One 7 (9), e43010. 10.1371/journal.pone.0043010 23028440 PMC3461009

[B46] Harmes K. M. Blackwood R. A. Burrows H. L. Cooke J. M. Harrison R. V. Passamani P. P. (2013). Otitis media: Diagnosis and treatment. Am. Fam. Physician 88 (7), 435–440. 24134083

[B47] Hernandez M. Leichtle A. Pak K. Webster N. J. Wasserman S. I. Ryan A. F. (2015). The transcriptome of a complete episode of acute otitis media. BMC Genomics 16 (1), 259. 10.1186/s12864-015-1475-7 25888408 PMC4394589

[B48] Igartua C. Davenport E. R. Gilad Y. Nicolae D. L. Pinto J. Ober C. (2017). Host genetic variation in mucosal immunity pathways influences the upper airway microbiome. Microbiome 5 (1), 16. 10.1186/s40168-016-0227-5 28143570 PMC5286564

[B49] Ilia S. Goulielmos G. N. Samonis G. Galanakis E. (2014). Polymorphisms in IL-6, IL-10, TNF-alpha, IFN-gamma and TGF-beta1 genes and susceptibility to acute otitis media in early infancy. Pediatr. Infect. Dis. J. 33 (5), 518–521. 10.1097/INF.0000000000000229 24463810

[B50] Imhann F. Vila A. V. Bonder M. J. Fu J. Gevers D. Visschedijk M. C. (2018). Interplay of host genetics and gut microbiota underlying the onset and clinical presentation of inflammatory bowel disease. Gut 67 (1), 108–119. 10.1136/gutjnl-2016-312135 27802154 PMC5699972

[B51] Jervis-Bardy J. Leong L. E. Marri S. Smith R. J. Choo J. M. Smith-Vaughan H. C. (2015). Deriving accurate microbiota profiles from human samples with low bacterial content through post-sequencing processing of illumina miSeq data. Microbiome 3, 19. 10.1186/s40168-015-0083-8 25969736 PMC4428251

[B52] Johnston J. Hoggard M. Biswas K. Astudillo-García C. Radcliff F. J. Mahadevan M. (2019). Pathogen reservoir hypothesis investigated by analyses of the adenotonsillar and middle ear microbiota. Int. J. Pediatr. Otorhinolaryngol. 118, 103–109. 10.1016/j.ijporl.2018.12.030 30599284

[B53] Kanmani P. Clua P. Vizoso-Pinto M. G. Rodriguez C. Alvarez S. Melnikov V. (2017). Respiratory commensal bacteria Corynebacterium pseudodiphtheriticum improves resistance of infant mice to respiratory syncytial virus and streptococcus pneumoniae superinfection. Front. Microbiol. 8, 1613. 10.3389/fmicb.2017.01613 28878760 PMC5572367

[B54] Kaur R. Adlowitz D. G. Casey J. R. Zeng M. Pichichero M. E. (2010). Simultaneous assay for four bacterial species including Alloiococcus otitidis using multiplex- PCR in children with culture negative acute otitis media. Pediatr. Infect. Dis. J. 29 (8), 741–745. 10.1097/INF.0b013e3181d9e639 20335823 PMC3581301

[B55] Kaur R. Casey J. R. Pichichero M. E. (2013). Relationship with original pathogen in recurrence of acute otitis media after completion of amoxicillin/clavulanate: bacterial relapse or new pathogen. Pediatr. Infect. Dis. J. 32 (11), 1159–1162. 10.1097/INF.0b013e31829e3779 23736142 PMC3845822

[B56] Kaur R. Casey J. R. Pichichero M. E. (2016). Emerging Streptococcus pneumoniae strains colonizing the nasopharynx in children after 13-valent Pneumococcal conjugate vaccination in comparison to the 7-valent era, 2006-2015. Pediatr. Infect. Dis. J. 35 (8), 901–906. 10.1097/INF.0000000000001206 27420806 PMC4948952

[B57] Kaur R. Morris M. Pichichero M. E. (2017). Epidemiology of acute otitis media in the postpneumococcal conjugate vaccine era. Pediatrics 140 (3), e20170181. 10.1542/peds.2017-0181 28784702 PMC5574724

[B58] Kerschner J. E. Hong W. Taylor S. R. Kerschner J. A. Khampang P. Wrege K. C. (2013). A novel model of spontaneous otitis media with effusion (OME) in the Oxgr1 knock-out mouse. Int. J. Pediatr. Otorhinolaryngol. 77 (1), 79–84. 10.1016/j.ijporl.2012.09.037 23200873 PMC3535456

[B59] Kozyrskyj A. Klassen T. P. Moffatt M. Harvey K. (2010). Short-course antibiotics for acute otitis media. Cochrane Database Syst. Rev. 9,CD001095. 10.1002/14651858.CD001095.pub2 PMC7052812 20824827

[B60] Krishnamurthy A. McGrath J. Cripps A. W. Kyd J. M. (2009). The incidence of Streptococcus pneumoniae otitis media is affected by the polymicrobial environment particularly Moraxella catarrhalis in a mouse nasal colonisation model. Microbes Infect. 11 (5), 545–553. 10.1016/j.micinf.2009.03.001 19306940

[B61] Krueger A. Val S. Pérez-Losada M. Panchapakesan K. Devaney J. Duah V. (2017). Relationship of the middle ear effusion microbiome to secretory mucin production in pediatric patients with chronic otitis media. Pediatr. Infect. Dis. J. 36 (7), 635–640. 10.1097/INF.0000000000001493 28027286

[B62] Kurilshikov A. Cisca W. Fu J. Zhernakova A. (2017). Host genetics and gut microbiome: challenges and perspectives. Trends Immunol. 38 (9), 633–647. 10.1016/j.it.2017.06.003 28669638

[B63] Kvaerner K. J. Tambs K. Harris J. R. Magnus P. (1997). Distribution and heritability of recurrent ear infections. Ann. Otol. Rhinol. Laryngol. 106 (8), 624–632. 10.1177/000348949710600802 9270423

[B64] Lamas B. Richard M. L. Leducq V. Pham H. P. Michel M. L. Da Costa G. (2016). CARD9 impacts colitis by altering gut microbiota metabolism of tryptophan into aryl hydrocarbon receptor ligands. Nat. Med. 22 (6), 598–605. 10.1038/nm.4102 27158904 PMC5087285

[B65] Lang A. Holt K. E. Inouye M. Holt P. G. Teo S. M. Tang H. H. (2019). Nasopharyngeal microbiome during health and illness in early life. J. Allergy Clin. Immunol. 141 (2), AB109. 10.1016/j.jaci.2017.12.348

[B66] Lao O. Lu T. T. Nothnagel M. Junge O. Freitag-Wolf S. Caliebe A. (2008). Correlation between genetic and geographic structure in Europe. Curr. Biol. 18 (16), 1241–1248. 10.1016/j.cub.2008.07.049 18691889

[B67] Lappan R. Imbrogno K. Sikazwe C. Anderson D. Mok D. Coates H. (2018). A microbiome case-control study of recurrent acute otitis media identified potentially protective bacterial genera. BMC Microbiol. 18 (1), 13. 10.1186/s12866-018-1154-3 29458340 PMC5819196

[B68] Lappan R. J. (2019). Using omics technologies to understand pathogenesis and seek alternative therapies for otitis media in children. 10.26182/5cf73acaee262.

[B69] Laufer A. Metlay J. Gent J. Fennie K. Kong Y. Pettigrew M. (2011). Microbial communities of the upper respiratory tract and otitis media in children. mBio 2 (1), e00245–e0010. 10.1128/mBio.00245-10 PMC3031303 21285435

[B70] Lee S. Lee E. Park Y. Hong S. (2018). Microbiome in the gut-skin axis in atopic dermatitis. Allergy Asthma Immunol. Res. 10 (4), 354–362. 10.4168/aair.2018.10.4.354 29949831 PMC6021588

[B71] Liang D. Leung R. Guan W. Au W. (2018). Involvement of gut microbiome in human health and disease: brief overview, knowledge gaps, and research opportunities. Gut Pathog. 10, 3. 10.1186/s13099-018-0230-4 29416567 PMC5785832

[B72] Lieberthal A. S. Carroll A. E. Chonmaitree T. Ganiats T. G. Hoberman A. Jackson M. A. (2013). The diagnosis and management of acute otitis media. Pediatrics 131 (3), e965–e999. 10.1542/peds.2012-3488 23439909

[B73] Lysaght A. C. Yuan Q. Fan Y. Kalwani N. Caruso P. Cunnane M. (2014). FGF23 deficiency leads to mixed hearing loss and middle ear malformation in mice. PloS One 9 (9), e107681. 10.1371/journal.pone.0107681 25243481 PMC4171482

[B74] MacArthur C. J. Wilmot B. Wang L. Schuller M. Lighthall J. Trune D. (2014). Genetic susceptibility to chronic otitis media with effusion: candidate gene single nucleotide polymorphisms. Laryngoscope 124 (5), 1229–1235. 10.1002/lary.24349 23929584 PMC3971016

[B75] Man W. van Dongen T. Venekamp R. Puimakers V. Chu M. van Houten M. (2019a). Respiratory microbiota predicts clinical disease course of acute otorrhea in children with tympanostomy tubes. Pediatr. Infect. Dis. J. 38 (6), e116–e125. 10.1097/INF.0000000000002215 30299424

[B76] Man W. H. van Houten M. A. Mérelle M. E. Vlieger A. M. Chu M. L. Jansen N. J. (2019b). Bacterial and viral respiratory tract microbiota and host characteristics in children with lower respiratory tract infections: a matched case-control study. Lancet Respir. Med. 7 (5), 417–426. 10.1016/S2213-2600(18)30449-1 30885620 PMC7172745

[B77] Marchisio P. Santagati M. Scillato M. Baggi E. Fattizzo M. Rosazza C. (2015). Streptococcus salivarius 24SMB administered by nasal spray for the prevention of acute otitis media in otitis-prone children. Eur. J. Clin. Microbiol. Infect. Dis. 34 (12), 2377–2383. 10.1007/s10096-015-2491-x 26385346

[B78] Mittal R. Lisi C. V. Gerring R. Mittal J. Mathee K. Narasimhan G. (2015). Current concepts in the pathogenesis and treatment of chronic suppurative otitis media. J. Med. Microbiol. 64, 1103–1116. 10.1099/jmm.0.000155 26248613 PMC4835974

[B79] Mittal R. Parrish J. M. Soni M. Mittal J. Mathee K. (2018). Microbial otitis media: recent advancements in treatment, current challenges and opportunities. J. Med. Microbiol. 67 (10), 1417–1425. 10.1099/jmm.0.000810 30084766

[B80] Mittal R. Debs L. H. Patel A. P. Nguyen D. Blackwelder P. Yan D. (2019). Otopathogenic Staphylococcus aureus invades human middle ear epithelial cells primarily through cholesterol dependent pathway. Sci Rep. 9, 10777. 10.1038/s41598-019-47079-7 31346200 PMC6658548

[B81] Neeff M. Biswas K. Hoggard M. Taylor M. W. Douglas R. (2016). Molecular microbiological profile of chronic suppurative otitis media. J. Clin. Microbiol. 54 (10), 2538–2546. 10.1128/JCM.01068-16 27487953 PMC5035421

[B82] Nokso-Koivisto J. Chonmaitree T. Jennings K. Matalon R. Block S. Patel J. A. (2014). Polymorphisms of immunity genes and susceptibility to otitis media in children. PloS One 9 (4), e93930. 10.1371/journal.pone.0093930 24718616 PMC3981756

[B83] Pettigrew M. M. Gent J. F. Pyles R. B. Miller A. L. Nokso-Koivisto J. Chonmaitree T. (2011). Viral-bacterial interactions and risk of acute otitis media complicating upper respiratory infection. J. Clin. Microbiol. 49 (11), 3750–3755. 10.1128/JCM.01186-11 21900518 PMC3209086

[B84] Pettigrew M. Laufer A. Gent J. Kong Y. Fennie K. Metlay J. (2012). Respiratory tract microbial communities, acute otitis media pathogens, and antibiotic use in healthy and sick children. Appl. Environ. Microbiol. 78 (17), 6262–6270. 10.1128/AEM.01051-12 22752171 PMC3416608

[B85] Pichichero M. E. Marsocci S. M. Murphy M. L. Hoeger W. Francis A. B. Green J. L. (2001). A prospective observational study of 5-, 7-, and 10-day antibiotic treatment for acute otitis media. Otolaryngol. Head Neck Surg. 124 (4), 381–387. 10.1067/mhn.2001.114311 11283494

[B86] Pichichero M. E. Casey J. R. Hoberman A. Schwartz R. (2008). Pathogens causing recurrent and difficult-to-treat acute otitis media, 2003-2006. Clin. Pediatr. (Phila) 47 (9), 901–906. 10.1177/0009922808319966 18559884

[B87] Pichichero M. (2009). Widening differences in acute otitis media study populations. Clin. Infect. Dis. 49 (11), 1648–1649. 10.1086/647934 19886802

[B88] Pickering J. Prosser A. Corscadden K. de Gier C. Richmond P. Zhang G. (2016). *Haemophilus haemolyticus* Interaction with Host Cells Is Different to Nontypeable *Haemophilus influenzae* and Prevents NTHi Association with Epithelial Cells. Front. Cell Infect. Microbiol. 6, 50. 10.3389/fcimb.2016.00050 27242968 PMC4860508

[B89] Rovers M. Haggard M. Gannon M. Koeppen-Schomerus G. Plomin R. (2002). Heritability of symptom domains in otitis media: a longitudinal study of 1,373 twin pairs. Am. J. Epidemiol. 155 (10), 958–964. 10.1093/aje/155.10.958 11994236

[B90] Rye M. S. Wiertsema S. P. Scaman E. S. Oommen J. Sun W. Francis R. W. (2011). FBXO11, a regulator of the TGFbeta pathway, is associated with severe otitis media in Western Australian children. Genes Immun. 12 (5), 352–359. 10.1038/gene.2011.2 21293382

[B91] Rye M. S. Warrington N. M. Scaman E. S. Vijayasekaran S. Coates H. L. Anderson D. (2012). Genome-wide association study to identify the genetic determinants of otitis media susceptibility in childhood. PloS One 7 (10), e48215. 10.1371/journal.pone.0048215 23133572 PMC3485007

[B92] Rye M. S. Wiertsema S. P. Scaman E. S. Thornton R. Francis R. W. Vijayasekaran S. (2013). Genetic and functional evidence for a role for SLC11A1 in susceptibility to otitis media in early childhood in a Western Australian population. Infect. Genet. Evol. 16, 411–418. 10.1016/j.meegid.2013.03.023 23538334

[B93] Sale M. M. Chen W. M. Weeks D. E. Mychaleckyi J. C. Hou X. Marion M. (2011). Evaluation of 15 functional candidate genes for association with chronic otitis media with effusion and/or recurrent otitis media (COME/ROM). PloS One 6 (8), e22297. 10.1371/journal.pone.0022297 21857919 PMC3156706

[B94] Santos-Cortez R. L. P. Chiong C. M. Reyes-Quintos M. R. T. Tantoco M. L. C. Wang X. Acharya A. (2015). Rare A2ML1 variants confer susceptibility to otitis media. Nat. Genet. 47 (8), 917–920. 10.1038/ng.33447 26121085 PMC4528370

[B95] Santos-Cortez R. L. P. Hutchinson D. S. Ajami N. J. Reyes-Quintos M. R. T. Tantoco M. L. C. Labra P. J. (2016). Middle ear microbiome differences in indigenous filipinos with chronic otitis media due to a duplication in the A2ML1 gene. Infect. Dis. Poverty 5, 97. 10.1186/s40249-016-0189-7 27799062 PMC5088646

[B96] Santos-Cortez R. L. P. Chiong C. M. Frank D. N. Ryan A. F. Giese A. P. J. Roberts T. B. (2018). FUT2 variants confer susceptibility to familial otitis media. Am. J. Hum. Genet. 103 (5), 679–690. 10.1016/j.ajhg.2018.09.010 30401457 PMC6217759

[B97] Schilder A. G. Chonmaitree T. Cripps A. W. Rosenfeld R. M. Casselbrant M. L. Haggard M. P. (2016). Otitis media. Nat. Rev. Dis. Primers 2, 16063. 10.1038/nrdp.2016.63 27604644 PMC7097351

[B98] Segade F. Daly K. A. Allred D. Hicks P. J. Cox M. Brown M. (2006). Association of the FBXO11 gene with chronic otitis media with effusion and recurrent otitis media: the Minnesota COME/ROM Family Study. Arch. Otolaryngol. Head Neck Surg. 132 (7), 729–733. 16847180 10.1001/archotol.132.7.729 PMC1904347

[B99] Shin S. Koh S. Woo C. Lim J. (2014). PAI-1 inhibits development of chronic otitis media and tympanosclerosis in a mouse model of otitis media. Acta Otolaryngol. 134 (12), 1231–1238. 10.3109/00016489.2014.940554 25399881

[B100] Siddiq S. Grainger J. (2015). The diagnosis and management of acute otitis media: American Academy of Pediatrics Guidelines 2013. Arch. Dis. Child Educ. Pract. Ed. 100, 193–197. 25395494 10.1136/archdischild-2013-305550

[B101] Sillanpää S. Kramna L. Oikarinen S. Sipilä M. Rautiainen M. Aittoneiemi J. (2017). Next-generation sequencing combined with specific PCR assays to determine the bacterial 16S rRNA gene profiles of middle ear fluid collected from children with acute otitis media. mSphere 2 (2), e00006–e00017. 10.1128/mSphere.00006-17 28357413 PMC5362748

[B102] Sokolowska M. Frei R. Lunjani N. Akdis C. A. O’Mahony L. (2018). Microbiome and asthma. Asthma Res. Pract. 4, 1. 10.1186/s40733-017-0037-y 29318023 PMC5755449

[B103] Tam V. Patel N. Turcotte M. Bosse Y. Pare G. Meyre D. (2019). Benefits and limitations of genome-wide association studies. Nat. Rev. Genet. 20 (8), 467–484. 10.1038/s41576-019-0127-1 31068683

[B104] Tateossian H. Morse S. Parker A. Mburu P. Warr N. Acevedo-Arozena A. (2013). Otitis media in the Tgif knockout mouse implicates TGFbeta signaling in chronic middle ear inflammatory disease. Hum. Mol. Genet. 22 (13), 2553–2565. 10.1093/hmg/ddt103 23459932 PMC3674796

[B105] Teo S. M. Mok D. Pham K. Kusel M. Serralha M. Troy N. (2015). The infant nasopharyngeal microbiome impacts severity of lower respiratory infection and risk of asthma development. Cell Host Microbe 17 (5), 704–715. 10.1016/j.chom.2015.03.008 25865368 PMC4433433

[B106] Tong H. H. Lambert G. Li Y. X. Thurman J. M. Stahl G. L. Douthitt K. (2014). Deletion of the complement C5a receptor alleviates the severity of acute pneumococcal otitis media following influenza A virus infection in mice. PloS One 9 (4), e95160. 10.1371/journal.pone.0095160 24740152 PMC3989264

[B107] Tremlett H. Bauer K. Appel-Cresswell S. Finlay B. Waubant E. (2017). The gut microbiome in human neurological disease: a review. Ann. Neurol. 81 (3), 369–382. 10.1002/ana.24901 28220542

[B108] Turnbaugh P. J. Hamady M. Yatsunenko T. Cantarel B. L. Duncan A. Ley R. E. (2009). A core gut microbiome in obese and lean twins. Nature 457 (7228), 480–484. 10.1038/nature07540 19043404 PMC2677729

[B109] Turpin W. Espin-Garcia O. Xu W. Silverber M. S. Kevans D. Smith M. I. (2016). Association of host genome with intestinal microbial compositionin a large healthy cohort. Nat. Genet. 48 (11), 1413–1417. 10.1038/ng.3693 27694960

[B110] Uehara Y. Nakama H. Agematsu K. Uchida M. Kawakami Y. Fattah A. S. M. (2000). Bacterial interference among nasal inhabitants: eradication of *Staphylococcus aureus* from nasal cavities by artificial implantation of *Corynebacterium* sp. J. Hosp. Infect. 44 (2), 127–133. 10.1053/jhin.1999.0680 10662563

[B111] Van Ingen G. Li J. Goedegebure A. Pandey R. Li Y. R. March M. E. (2016). Genome-wide association study for acute otitis media in children identifies FNDC1 as disease contributing gene. Nat. Commun. 7, 12792. 10.1038/ncomms12792 27677580 PMC5052699

[B112] Walker R. E. Walker C. G. Camargo C. A. Bartley J. Flint D. Thompson J. M. D. (2019). Nasal microbial composition and chronic otitis media with effusion: A case-control study. PloS One 14 (2), e0212473. 10.1371/journal/pone/0212473 30794625 PMC6386383

[B113] Wang W. Zhou A. Zhang X. Xiang Y. Huang Y. Wang L. (2014). Interleukin 17A promotes pneumococcal clearance by recruiting neutrophils and inducing apoptosis through a p38 mitogen-activated protein kinase dependent mechanism in acute otitis media. Infect. Immun. 82 (6), 2368–2377. 10.1128/IAI.00006-14 24664502 PMC4019148

[B114] Wang J. Thingholm L. B. Skiecevičiené J. Rausch P. Kummen M. Hov J. R. (2016). Genome-wide association analysis identifies variation in vitamin D receptor and other host factors influencing the gut microbiota. Nat. Genet. 48 (11), 1396–1406. 10.1038/ng.3695 27723756 PMC5626933

[B115] Weissbrod O. Rothschild D. Barkan E. Segal E. (2018). Host genetics and microbiome associations through the lens of genome wide association studies. Curr. Opin. Microbiol. 44, 9–19. 10.1016/j.mib.2018.05.003 29909175

[B116] Yatsunenko T. Rey F. E. Manary M. J. Trehan I. Dominguez-Bello M. G. Contreras M. (2012). Human gut microbiome viewed across age and geography. Nature 486 (7402), 222–227. 10.1038/nature11053 22699611 PMC3376388

[B117] Yoshimura A. Wakabayashi Y. Mori T. (2010). Cellular and molecular basis for the regulation of inflammation by TGF-beta. J. Biochem. 147 (6), 781–792. 10.1093/jb/mvq043 20410014 PMC2912031

[B118] Zhang Y. Yu H. Xu M. Han F. Tian C. Kim S. (2012). Pathological features in the LmnaDhe/+ mutant mouse provide a novel model of human otitis media and laminopathies. Am. J. Pathol. 181 (3), 761–774. 10.1016/j.ajpath.2012.05.031 22819531 PMC3432432

